# Financial Strain, Parental Smoking, and the Great Recession: An Analysis of the UK Millennium Cohort Study

**DOI:** 10.1093/ntr/ntw269

**Published:** 2016-10-19

**Authors:** Caoimhe S McKenna, Catherine Law, Anna Pearce

**Affiliations:** 1 Population, Policy and Practice, Great Ormond Street Institute of Child Health, University College London (UCL), London, UK

## Abstract

**Introduction:**

During the recent “Great Recession,” many families in the United Kingdom experienced increased financial strain (FS). The aim of this study was to determine if increases in FS, occurring over the period of the “Great Recession,” were associated with increased risks of persistent and relapsed tobacco use among parents.

**Methods:**

We analyzed the Millennium Cohort Study, a longitudinal study of 18819 children born in the United Kingdom between 2000 and 2002. Surveys at 7 (T1, 2008) and 11 years (T2, 2012) spanned the “Great Recession.” Three measures of increased FS were defined; “became income poor” (self-reported household income dropped below the “poverty line” between T1 and T2); “developed difficulty managing” (parental report of being “financially comfortable” at T1 and finding it “difficult to manage” at T2); “felt worse off” (parental report of feeling financially “worse off” at T2, compared to T1). Poisson regression was used to estimate risk ratios (*RR*), adjusted *RR*s (*aRR*), and 95% confidence intervals for three outcomes: “persistent tobacco use,” “new reported tobacco use,” and “relapsed tobacco use.”

**Results:**

Parents in households which “became income poor” over the period of the “Great Recession” were significantly more likely to report “persistent tobacco use” (*aRR* = 2.17 [1.83–2.57]) or “new reported tobacco use” (*aRR* = 1.72 [1.04–2.83]). Ninety-five percent of “new reported tobacco users” had evidence of prior tobacco use suggesting the majority were “relapsed tobacco users.” Similar patterns were seen for those who “developed difficulty managing” and “felt worse off.”

**Conclusions:**

Increased tobacco use among financially strained families has the potential to widen inequalities and undermine the public health policies that have had positive impacts on tobacco consumption in the United Kingdom.

**Implications:**

While several studies have shown that FS is associated with a higher prevalence of tobacco use, heavier smoking, and relapsed tobacco use, most of this work used cross-sectional data and none has focused on parents. We used longitudinal data from the UK Millennium Cohort Study, between 2008 and 2012, to examine the association between FS and parental smoking. We show that parents who experienced increased FS, over the period of the “Great Recession,” were more likely to continue using tobacco or to relapse.

## Introduction

Financial strain (FS) occurs when resources are inadequate to meet needs and/or expectations. FS has been associated with a higher prevalence of tobacco use and heavier smoking.^1,2^ FS also appears to hinder smoking cessation and increase the likelihood of relapse.^3–5^ Siahpush et al.^6^ found that smokers experiencing FS were more keen to quit smoking but were less likely to be successful.

The “tension-reduction hypothesis”^7,8^ proposes that tobacco is used to relieve the negative emotions resulting from stress exposure. Qualitative research by Graham^9^ suggested the major reasons for relapse among a sample of lower socioeconomic mothers, who had previously given up smoking, were difficulty coping with everyday problems, stress, and financial pressures.

In 2008, the United Kingdom entered “technical economic recession” in the context of a global financial crisis. The years following were characterized by rising unemployment, a fall in real wages, and rising levels of absolute poverty.^10–13^ This is a time when many families in the United Kingdom experienced an increase in FS. Literature examining tobacco use among adults, following the “Great Recession,” found that smoking was more common in those experiencing increased FS^14^ and inequalities in smoking increased.^15^ However, no study has focused on parents. Parental smoking is associated with higher rates of respiratory disorders among children,^16,17^ and those with a smoking parent are more likely to initiate smoking as adolescents.^18,19^ The pressures of providing for dependent family members during times of economic hardship might increase FS more for parents than for other adults, and this may impact on smoking behavior.

The aim of this study was to determine if increases in household FS (based on parental perception and also changes in household income), occurring over the period of the “Great Recession,” were associated with increased risks of persistent or relapsed tobacco use among parents.

## Methods

We examined data from the Millennium Cohort Study (MCS), a longitudinal study of children born in the United Kingdom between 2000 and 2002. The original sample included 18296 singleton children. To date, MCS data are available for analysis at age 9 months, 3 years, 5 years, 7 years, and 11 years. The information collected includes a wide range of parental-reported sociodemographic and health factors (more information on the MCS can be found at www.cls.ioe.ac.uk/MCS). Surveys carried out when the MCS children were aged 7 (T1, 2008) and 11 years (T2, 2012) spanned the period of the “Great Recession.” At age 11 years, 69.7% (*n* = 13112) of the original sample took part. This included 11387 natural mothers (*n* = 11220) and fathers (*n* = 167) who were the same main respondent at T1 and T2. This was our main working sample. Prior smoking history was also assessed for 9640 natural mothers or fathers who had been the same main respondent at all prior sweeps, in a subanalysis.

### Exposure: Increased FS

Three measures of increased FS between ages 7 (T1) and 11 years (T2) were defined, each capturing different aspects of FS.

#### Became Income Poor

Household income was ≥60% of contemporary median at T1 (ie, above the poverty line) and <60% of contemporary median at T2 (ie, below the poverty line). Incomes were reported by parents and equivalized according to Organization for Economic Cooperation and Development (OECD) scales.^20^ The comparator group were those who “stayed nonpoor” (ie, above the “poverty line” at both T1 and T2).

#### Developed Difficulty Managing

Main respondents were asked at T1 and T2, “How well would you say you are managing financially these days?”. Possible responses were (1) living comfortably, (2) doing alright, (3) just about getting by, (4) finding it quite difficult, and (5) finding it very difficult. An increase in household FS was defined as going from a score of 1–3 at T1 to 4/5 at T2. In the analyses, the comparator group were those who “did not report difficulty managing” (ie, a score of 1–3 at both timepoints).

#### Felt Worse off

Main respondents were asked at T2, “Compared with the time of the last interview would you say that you are better or worse off financially or about the same?”. Possible answers included (1) a lot better off, (2) a little better off, (3) about the same, (4) a little worse off, and (5) a lot worse off. An increase in FS was defined as stating you were “a little” or “a lot worse off,” compared with T1. The comparator group were those who felt their finances were “about the same.”

Households which remained financially strained, according to our definitions, at T1 and T2 or moved out of FS at T2 were excluded from the analyses. All measures of increased FS were based on parental report.

### Outcomes: Tobacco Use

Main respondents were asked at T1 and T2, “Do you use tobacco products such as cigarettes, cigars, a pipe or chewing tobacco at all nowadays?”. The sample was limited to natural parents who had been the same respondent at both T1 and T2 to ensure consistency.

Those who reported tobacco use at both timepoints were considered “persistent tobacco users.” “New reported tobacco use” was defined as parental report of tobacco use at T2, which was not reported at T1. The majority of “new reported tobacco use” occurred in parents who had an identifiable history of tobacco use at interviews prior to T1. We therefore carried out an additional analysis examining “relapsed tobacco use.”

In all analyses, the reference group was those who did not use tobacco at both timepoints and those who “gave up” between T1 and T2. Less than 1% were missing tobacco use data at T1 or T2.

### Statistical Analysis

Poisson regression was used to estimate unadjusted and adjusted risk ratios and 95% confidence intervals^21^ for “persistent,” “new,” and “relapsed smoking” according to the three measures of FS. We adjusted our analyses for lone parenthood (one parent household at T1), ethnicity (white British/Irish, other), maternal level of education at 9 months (degree level or above), and parental age at T1 (continuous variable, years).

Analyses were conducted in Stata/SE 13 (Stata Corporation, College Station, TX), using “svy” commands to account for clustered sampling design and attrition. Data were downloaded from the UK Data Service, University of Essex, and University of Manchester, in April 2014.

## Results

Twenty-eight percent (*n* = 2905) of main respondents reported tobacco use at T1, and 25% (*n* = 2614) of the main respondents reported tobacco use at T2. Table 1 summarizes the baseline demographics of tobacco users and nonusers at T1.

**Table 1.** Baseline (T1) Demographics of Tobacco Users and Nontobacco Users (at T1)

	Tobacco users	Nontobacco users
	*n* = 2905	*n* = 8447
Demographics	*n* (%)/average (95% CI)	*n* (%)/average (95% CI)
Mean age of main respondent (y)	34.9 (33.8–34.2)	37.0 (36.9–37.2)
Ethnicity British/Irish white	2336 (93.7%)	6394 (85.0%)
Mother degree-level education or higher^a^	144 (3.8%)	1999 (21.16%)
Anyone in the household employed	2392 (82.8%)	7567 (89.1%)
Lone parent household	1053 (37.4%)	1219 (16.5%)
Mean number of children in household	2.6 (2.53–2.60)	2.54 (2.50–2.54)
Living in England	1702 (79.4%)	5583 (82.9%)
Main respondent natural mother	2840 (97.7%)	8346 (98.6%)

CI = confidence interval. Main respondents limited to natural mothers and fathers who took part at T1 and T2 (ie, main working sample). Percentages are survey weighted. Missing data (total sample = 11387): respondent age: *n* = 0; ethnicity: *n* = 1201; maternal education: *n* = 374; employment: *n* = 57; lone parenthood: *n* = 0; number of children: *n* = 1; residence: *n* = 0; tobacco use at T1: *n* = 35.

^a^Maternal level of education as reported when participant child was aged 9 months.

At T1, 29% (*n* = 3176) of main respondents were below the poverty line (“income poor”), and 43% (*n* = 4674) reported difficulty managing financially. At T2, 19% (*n* = 2070) of main respondents were below the poverty line, 47% (*n* = 5195) reported difficulty managing financially, and 36% (*n* = 4059) felt worse off.

Between T1 and T2, 39.2% (*n* = 5206/13005) of all households experienced an increase in FS. Those who “became income poor” made up the smallest proportion (9.4%), and those who “felt worse off” made up the largest (89.6%).

Main respondents in households which experienced an increase in FS between T1 and T2 were significantly more likely to report “persistent tobacco use” between T1 and T2 than those who did not report FS, regardless of the measure of FS examined (Table 2, column A).

**Table 2.** *RR*s, *aRR*s, and 95% CIs for Tobacco Use at T2, New Reported Tobacco Use Between T1 and T2, and “Relapsed” Tobacco Use Among Respondents Who Experienced an Increase in Financial Strain Between T1 (7 Years) and T2 (11 Years)

Measures of increased financial strain and comparator groups	Outcomes								
	(A) Persistent tobacco use (T1–T2)^a^			(B) New reported tobacco use (T1–T2)^a^			(C) “Relapsed” tobacco use^a^		
	(*n* = 2252/10952)			(*n* = 362/9042)			(*n* = 282/8962)		
	% (*n*)	*RR* (CI)	*aRR* (CI)	% (*n*)	*RR* (CI)	*aRR* (CI)	% (*n*)	*RR* (CI)	*aRR* (CI)
Became income poor	43.0 (184)	2.96*** (2.56, 3.43)	2.17*** (1.83, 2.57)	9.0 (23)	2.74*** (1.77, 4.22)	1.72* (1.04, 2.83)	5.6 (15)	2.03** (1.20, 3.44)	1.57 (0.88, 2.77)
Stayed nonpoor	14.5 (981)	—	—	3.3 (213)	—	—	2.7 (181)	—	—
Developed difficulty managing	34.7 (317)	1.86*** (1.65, 2.09)	1.54*** (1.36, 1.75)	8.0 (49)	2.15*** (1.55, 2.99)	1.81** (1.25, 2.63)	5.7 (36)	1.96** (1.31, 2.94)	1.77** (1.18, 2.69)
Did not report difficulty managing	18.7 (1468)	—	—	3.7 (255)	—	—	2.9 (203)	—	—
Felt “worse off”	24.5 (880)	1.13* (1.02, 1.26)	1.15* (1.03, 1.28)	4.9 (148)	1.33* (1.02, 1.75)	1.32* (1.01, 1.74)	3.6 (112)	1.23 (0.92, 1.66)	1.25 (0.93, 1.66)
Felt “the same”	21.6 (773)	—	—	3.7 (106)	—	—	2.9 (84)	—	—
All % (CI)	22.5 (22.1, 24.0)	—	—	4.3 (3.8, 4.8)	—	—	3.3 (2.9, 3.8)	—	—

*aRR* = adjusted risk ratio; CI = confidence intervals; *RR* = risk ratio. Percentages are survey weighted to account for study design and attrition.^20^

(A) Parent reported tobacco use at T1 and T2 versus those who did not use tobacco at both time points and those who “gave up” between T1 and T2. (B) Parent report of tobacco use at T2, which was not reported at T1 versus those who did not use tobacco at both time points and those who “gave up” between T1 and T2. (C) Parent report of tobacco use at T2, which was not reported at T1, and for whom there was a known history of prior tobacco use (ie, “relapsed tobacco users”) versus those who did not use tobacco at both timepoints and those who “gave up” between T1 and T2.

^a^Main respondents were limited to natural mothers and fathers to ensure the main respondent was the same at both T1 and T2. *RR*s are adjusted for lone parenthood (one parent household at age 7 years), ethnicity (main respondent white British/Irish, other), maternal level of education at 9 months (degree level or above), and parental age (continuous variable, years). Missing data: lone parenthood: *n* = 0; ethnicity: *n* = 1201; maternal education: *n* = 374; and parental age: *n* = 0.

*p < .05, **p < .01, ***p < .001.

Main respondents who experienced an increase in FS were also significantly more likely to report tobacco use at T2, which was not reported at T1 (“new reported tobacco use”) (Table 2, column B). Ninety-five percent (*N* = 282/296) of these “new reported tobacco users” had evidence of prior tobacco use based on data from MCS interviews prior to T1, suggesting that the majority were “relapsed users.” The association between FS stain and relapsed tobacco use was similar to that seen for new tobacco use (Table 2, column C). Risk ratios remained elevated after adjustment for confounding factors (Table 2).

## Discussion

The findings of this nationally representative UK cohort support the hypothesis that parents who experienced increased FS over the period of the Great Recession were more likely to continue using tobacco and to relapse. The most probable explanation for this is that tobacco is a commonly used, affordable palliative for stress.^22^

The findings suggest that tobacco consumption is likely to increase among parents, during times of widespread economic hardship. This has the potential to undermine public health policies that have had positive impacts on tobacco consumption in the United Kingdom, such as taxation,^23^ plain packaged cigarettes,^24^ and the “smoking ban.”^25^

The findings are consistent with previous research showing a positive association between FS and tobacco use.^2,3,5^ While these studies focused on adults in general, our study has focused on parents. As parental smokers risk not only their own health, but that of their children, they are a particularly important subgroup of tobacco users.

Survey weights were used in the analyses to account for sampling design and attrition. However, parents who were lost to follow-up were significantly more likely to be tobacco users or to have a prior history of tobacco use (data not shown), and it is possible that this bias has not been fully accounted for. Parental report of smoking may also have been underestimated^26^; if under-reporting was more common in one group this may have biased the results.^27^ There may also be other explanations as to why parents reported new tobacco use between T1 and T2, for example the end of a pregnancy. There is also the potential for reverse causality. Siahpush et al.^28^ reported that households which contain smokers are more likely to develop FS, regardless of income. The measures of changes in FS were derived from reported variables, and no other source of information was available. “Difficultly managing financially” and “feeling worse off” are subjective measures, and it was not possible to determine if these measures accurately reflect the reality of household finances. Household income was also self-reported, although income poverty was defined according to standard cutoffs. Although our main working sample consisted predominantly of mothers, we retained main respondents who were natural fathers in our analysis as they may be in the role of the main carer. The generalizability of our results to fathers, who were not main respondents, may be limited. For 80 parents who had “new reported tobacco use,” it was not possible to identify a history of prior tobacco use in the dataset. This may be because parents had taken up tobacco use for the first time between T1 and T2 or that they had smoked previously but data on prior smoking was not available in the dataset.

Tobacco use is unequivocally bad for parental and child health. Parents experiencing increases in FS may find it more difficult to quit tobacco use; in addition, those who have previously quit may be more likely to relapse. Measures to buffer families from FS or increased investment in smoking cessation, during times of widespread FS such as economic recessions, may help reduce this risk.

## Funding

Research at the UCL Institute of Child Health and Great Ormond Street Hospital for Children receives a proportion of the funding from the Department of Health’s National Institute for Health Research Biomedical Research Centres funding scheme. The Millennium Cohort Study is funded by grants to former and current directors of the study from the Economic and Social Research Council (Professor Heather Joshi, Professor Lucinda Platt, and Professor Emla Fitzsimons) and a consortium of government funders. AP was funded by a Medical Research Council fellowship (MR/J012351/1). CMK was funded by a NIHR Academic Clinical Fellowship. All researchers were independent of the funders. The funders, original data creators, depositors, copyright holders, and data funders played no part in the study design, analysis or interpretation of the data, writing the report, or the decision to submit for publication.

## Declaration of Interests

*None declared*.
